# The short-term safety and efficacy of TANDEM microspheres of various sizes and doxorubicin loading concentrations for hepatocellular carcinoma treatment

**DOI:** 10.1038/s41598-021-91021-9

**Published:** 2021-06-10

**Authors:** Chia-Ying Lin, Yi-Sheng Liu, Kuang-Tse Pan, Chia-Bang Chen, Chein-Fu Hung, Chen-Te Chou

**Affiliations:** 1 Department of Medical Imaging, National Cheng Kung University Hospital, College of Medicine, National Cheng Kung University, No. 138 Sheng Li Road, Tainan, 704 Taiwan, ROC; 2 Department of Imaging and Intervention, Chang Gung Medical Foundation—Chang Gung Memorial Hospital, Linkou, No.5 FuXing Street, GueiShan, TaoYuan County 333 Taiwan, ROC; 3 Department of Radiology, Chang-Hua Christian Hospital, No. 135, Nan-Xiao Street, Changh-Hua City, Changhua County 500 Taiwan, ROC; 4 School of Medicine, Kaohsiung Medical University, Kaohsiung City, Taiwan, ROC; 5 Department of Molecular Biotechnology, College of Biotechnology and Bioresources, Dayeh University, Changhua City, Taiwan, ROC

**Keywords:** Cancer, Cancer therapy, Gastrointestinal cancer

## Abstract

Drug-eluting bead transarterial chemoembolization (DEB-TACE) is the most common treatment for unresectable hepatocellular carcinoma (HCC). However, the effect of drug loading concentration and microsphere size on treatment outcomes remains unclear. This retrospective study compares the outcomes of 87 HCC patients who underwent DEB-TACE with half-loaded or full-loaded doxorubicin (maximum capacity 50 mg/mL) in 75-µm or 100-µm microspheres. Treatment with 100-μm microspheres resulted in significantly lower rates of procedure-related complications (6.6% vs. 26.9%; P < 0.05), post-embolization syndrome (32.8% vs. 61.5%, P < 0.05), SIR complications (32.8% vs. 61.5%; P < 0.01) and adverse events involving abdominal pain (19.7% vs. 42.3%; P < 0.05). Half-load doxorubicin microspheres resulted in greater treatment response (OR, 4.00; 95% CI 1.06–15.13; P, 0.041) and shorter hospital stays (OR, − 1.72; 95% CI − 2.77–0.68; P, 0.001) than did microspheres loaded to full capacity. Stratified analysis further showed that patients treated with 100-μm half-load doxorubicin microspheres had a higher CR (63.6% vs 18.0%) and ORR (90.9 vs 54.0%) and a shorter hospital stay (1.6 ± 1.3 vs 4.2 ± 2.3 days) than did those treated with full-load microspheres (P < 0.05). Thus, the drug-loading concentration of microspheres in DEB-TACE should be carefully considered.

## Introduction

Liver cancer is the fourth leading cause of cancer-related deaths and sixth most common cancer in the world, with high prevalence in East Asia, South-Eastern Asia, and Northern and Western Africa^1,2^. The most common histological types of primary liver cancer are hepatocellular carcinoma (HCC) (80%) and intrahepatic cholangiocarcinoma (iCCA) (10–15%)^3^. Although considerable effort has been made to develop approaches for the prevention, monitoring, early detection, diagnosis, and treatment of liver cancer, HCC remains the main cause of cancer-related deaths in many parts of the world^4,5^.

Surgery, radiofrequency ablation, and liver transplantation are the standard curative treatments for early-stage. For patients diagnosed with intermediate-stage HCC, the Barcelona Clinic Liver Cancer (BCLC) guidelines recommend transarterial chemoembolization (TACE) as the gold standard treatment^6,7^. Conventional TACE (cTACE) selectively obstructs the tumor-feeding arteries by delivery of a chemotherapy drug loaded in lipiodol, resulting in tumor cytotoxicity and ischemic necrosis. Although several randomized control trials indicate survival benefits of cTACE for unresectable HCC^8–11^, cTACE has shortcomings that include reduced effectiveness of chemotherapeutic agents due to lipiodol effects, ineffective and unsustained release of chemotherapeutic agents, and heterogeneity in clinical practice and therapeutic schedules^12^.

To improve upon the disadvantages of cTACE, drug-eluting beads (DEB) have been developed. DEB-TACE provides sustained drug release, increased concentrations of cytotoxic agents in tumors, and lower toxicity to non-targeted tissues^13^. Randomized controlled trials and retrospective studies have demonstrated that DEB-TACE results in a better treatment response, longer survival, fewer adverse events, and delayed tumor progression compared to cTACE^14–19^. On the other hand, the size of the beads and loading dose of the drug may also affect the DEB-TACE treatment response, survival outcomes, and adverse events. The European Conference on Interventional Oncology in Florence recommends the use of 100–300 μm beads and a loading dose of 25–37.5 mg doxorubicin/mL of beads for HCC treatment^20^. Several studies report that treatment using small beads (100–300 μm) is associated with a better treatment response and survival outcome as well as fewer complications than that using large beads (300–700 μm)^21,22^. Moreover, the use of smaller beads m resulted in greater toxicity and adverse events^23,24^. It is likely that smaller beads can penetrate more deeply into smaller blood vessels with a higher spatial density, allowing more drugs to be distributed to deeper tumor and non-tumor tissues^25,26^. However, other studies report no significant difference in clinical outcomes between beads of size 70–150 μm and 100–300 μm^27^.

TANDEM drug-eluting microspheres contain negatively-charged sodium polymethacrylate that can interact with positively-charged chemotherapeutic drugs^28^. TANDEM microspheres have a tight and uniform size distribution of 40 ± 10 µm, 75 ± 15 µm, and 100 ± 25 µm. The recommended maximum dose of doxorubicin and irinotecan is 50 mg/mL. Several recent randomized controlled trials and retrospective cohort studies have shown the clinical values of drug-loadable TANDEM using microspheres of different sizes with improved pharmacokinetic characteristics^29–31^. However, most studies have used microspheres of a specific diameter range or two sizes simultaneously for HCC treatment^32,33^. Whether single-diameter 75-μm TANDEM microspheres are more effective and safer than 100-μm microspheres is unknown. Therefore, the aim of this study is to compare the safety and efficacy of DEB-TACE treatment for HCC using 75- and 100-μm TANDEM microspheres with different loading volumes of doxorubicin.

## Methods

### Study design

This retrospective study was conducted in accordance with the Declaration of Helsinki, and all participants provided written informed consent. The study was approved by the Institutional Review Board of National Cheng Kung University Hospital (No. B-ER-106-204). The inclusion criteria were as follows: (1) age > 18 years; and (2) pathological diagnosis of HCC. Patients with early/intermediate HCC should be deemed unsuitable for percutaneous or surgical ablation treatment. Patients received DEB-TACE treatment using doxorubicin-loaded 75- or 100-µm TANDEM microspheres (CeloNova Biosciences/Boston Scientific, Marlborough, MA, USA). A total of 87 HCC patients who underwent TACE with 75- or 100-µm microspheres between November 2014 and July 2016 were enrolled in this study and included in the analysis. Of these patients, 46 were TACE naïve and 42 had previously received TACE treatment. Of these patients, 40 were in a well-defined high-risk population (chronic hepatitis B infection or liver cirrhosis by any cause), with HCC confirmed by CT/MRI images (arterial phase hyperenhancement followed by portal venous phase or delayed washout appearance) and lab examinations (alpha-fetoprotein elevation). The other 47 cases of HCC were proven by means of biopsy. All patients underwent contrast-enhanced CT and MRI one month after TANDEM TACE embolization and every 3 months thereafter. Follow-up CT and MRI examination were used to determine the tumor response at 3 months after TACE treatment.

### DEB-TACE procedure

The TACE procedure was performed in a manner similar to that of previous reports^30,34–37^. Doxorubicin was loaded into TANDEM microspheres (CeloNova, 75 or 100 µm in diameter) according to the manufacturer’s instruction. TANDEM microspheres (75 µm and 100 µm) were loaded with doxorubicin to 50 mg/ml (full-loaded) or 25–35 mg/ml (half-loaded) and then injected in segmental or subsegmental hepatic artery. The use of antibiotic prophylaxis, analgesic, or antiemetic medications during chemoembolization procedures were at the physician’s discretion. Before chemoembolization, angiography of the hepatic arteries, mesenteric arteries and extrahepatic pathological branches was performed to confirm the anatomical eligibility and determine tumor feeder arteries. If “near stasis” was not achieved in the artery after first dose of doxorubicin/TANDEM microspheres, an additional volume of doxorubicin/TANDEM microspheres was injected.

### Evaluation of safety and efficacy objectives

Safety was assessed by monitoring the laboratory values for aspartate aminotransferase (AST), alanine aminotransferase (ALT), total bilirubin (T-bil), platelet (PLT), and creatinine (CRE) before and after TACE treatment. The occurrence of procedure-related complications, including bile duct injury, hepatic abscess, gastric mucosa injury, pulmonary embolism, and acute pancreatitis, was analyzed. In addition, the grade of post-embolization syndromes was recorded based on the guideline by Leung et al.^38^, while the SIR classification for complications was recorded based on the guideline of the Society of Interventional Radiology^39,40^. Adverse events were recorded and classified according to the Cancer Institute Common Terminology Criteria for Adverse Events version 5.0 (CTCAE). Adverse events included abdominal pain, fever, acute gastritis, reflux esophagitis, nausea, and liver abscess. Tumor response was assessed according to the modified Response Evaluation Criteria in Solid Tumors (mRECIST). In addition, the length of hospital stay was assessed and used as the treatment outcome.

### Statistical analysis

Data for continuous variables are presented as the mean ± standard deviation (SD) or median with inter-quartile range (IQR). Data for categorial variables are presented as the number and percentage. The Mann–Whiney test was performed to analyze differences between TANDEM microspheres sizes (75 µm vs. 100 µm) and doxorubicin-loading amount (full-loaded and half-loaded) in terms of variables regarding clinical characteristics, tumor burden, treatment outcomes, and adverse events. Fisher’s exact test was used to analyze categorical variables. For biochemical variables measured before and after TACE treatment, the Wilcoxon signed rank test was used to compare the measurements at two time points for each patient, and the differences from baseline were examined using the Mann–Whitney test. Logistic regression analysis was performed to examine the association between better treatment response and TANDEM size or doxorubicin-loading concentration. In this study, a better treatment response was defined as complete response. A linear regression model was used to analyze the length of hospital stays. The regression coefficients indicate the difference in days of hospital stay between TANDEM sizes and doxorubicin-loading concentrations. To examine the associations of TANDEM sizes and doxorubicin-loading concentration with overall survival (OS), Cox proportional hazards regression analysis was performed. Kaplan–Meier survival curves were constructed for TANDEM size and doxorubicin-loading concentration, and the log-rank test was performed to compare the survival curves. All regression analyses were adjusted for age, sex, clinical characteristics, tumor burden, and total TACE doxorubicin dosage. The significance level was set as two-sided p < 0.05. All statistical analyses were performed using SAS version 9.4 (Windows NT version, SAS Institute, Inc., Cary, NC, USA).

## Results

### Demographics and tumor characteristics

The demographic and tumor characteristics of the 87 patients who met the inclusion/exclusion criteria are shown in Table 1. The mean age was 67.2 years, and most patients were male (79.3%). Those patients testing positive for hepatitis virus included 41 with hepatitis B (HBV) infection (47.1%) and 26 with hepatitis C (HCV) infection (29.9%). Most of these patients did not receive medication for HBC or HCV. Most patients in the cohort had non-alcoholic liver disease (89.7%), non-fatty liver disease (82.8%), or non-infiltrative HCC (91.95%), and classified as ECOG 0 (96.6%). The median tumor diameter and Child–Pugh scores were 4.5 cm (range, 2.5–7.2) and 5 (range, 5–6), respectively. BCLC A classification was observed for 29.9% of the patient cohort. The total doxorubicin dosage for TACE was 47.5 ± 23.7 mg. Patients were divided into subgroups based on TANDEM size (75 µm or 100 µm) or doxorubicin-loading concentration (full-loaded and half-loaded). No significant difference was observed in the demographic or tumor characteristics between patients treated with 75-μm or 100-μm TANDEM microspheres or between patients treated with microspheres loaded to half or full capacity with doxorubicin (P > 0.05). In addition, there was no statistically significant difference in the total dosage of doxorubicin delivered between patients treated with 75- or 100-μm TANDEM microspheres (P, 0.144) or between patients treated with half-loaded and full-loaded doxorubicin (P, 0.675).

**Table 1:** Baseline demographics and tumor characteristics of HCC patients.

	Total (n = 87)	Tandem size			*P*-value^a^	Doxorubicin loading		*P*-value^a^
		75 μm (n = 26)	100 μm (n = 61)			Half-loaded (n = 15)	Full-loaded (n = 72)	
**Clinical characteristics**								
Age (years)^b^	67.2 ± 10.6	67.2 ± 9.4	67.2 ± 11.1		0.993	71.9 ± 8.0	66.3 ± 10.8	0.056
**Gender**					0.249			0.502
Male	69 (79.3%)	23 (88.5%)	46 (75.4%)			11 (73.3%)	58 (80.6%)	
Female	18 (20.7%)	3 (11.5%)	15 (24.6%)			4 (26.7%)	14 (19.4%)	
**Family medical history**					0.078			1.000
No	83 (95.4%)	23 (88.5%)	60 (98.4%)			15 (100%)	68 (94.4%)	
Yes	4 (4.6%)	3 (11.5%)	1 (1.6%)			0 (0%)	4 (5.6%)	
**HBV**					0.244			1.000
No	46 (52.9%)	11 (42.3%)	35 (57.4%)			8 (53.3%)	38 (52.8%)	
Yes	41 (47.1%)	15 (57.7%)	26 (42.6%)			7 (46.7%)	34 (47.2%)	
**HCV**					0.801			0.537
No	61 (70.1%)	19 (73.1%)	42 (68.9%)			12 (80.0%)	49 (68.1%)	
Yes	26 (29.9%)	7 (26.9%)	19 (31.2%)			3 (20.0%)	23 (31.9%)	
**Medication for HBV or HCV**					0.606			0.539
No	62 (71.3%)	20 (76.9%)	42 (68.8%)			12 (80.0%)	50 (69.4%)	
Yes	25 (28.7%)	6 (23.1%)	19 (31.2%)			3 (20.0%)	22 (30.6%)	
**Fatty liver disease**					1.000			0.717
No	72 (82.8%)	22 (84.6%)	50 (82.0%)			12 (80.0%)	60 (83.3%)	
Yes	15 (17.2%)	4 (15.4%)	11 (18.0%)			3 (20.0%)	12 (16.7%)	
**Alcoholic liver disease**					0.719			0.650
No	78 (89.7%)	24 (92.3%)	54 (88.5%)			13 (86.7%)	65 (90.3%)	
Yes	9 (10.3%)	2 (7.7%)	7 (11.5%)			2 (13.3%)	7 (9.7%)	
**Tumor burden**								
Tumor size^c^	4.5 (2.5, 7.2)	4.4 (2.1, 8.6)	4.7 (2.6, 7.2)		0.817	3.5 (2.2, 6.6)	5 (2.7, 7.5)	0.212
Child–Pugh score^c^	5 (5, 6)	5 (5, 6)	5 (5, 6)		0.231	6 (5, 7)	5 (5, 6)	0.010
**ECOG**					1.000			1.000
0	84 (96.6%)	25 (96.2%)	59 (96.7%)			15 (100%)	69 (95.8%)	
1	3 (3.5%)	1 (3.8%)	2 (3.3%)			0 (0%)	3 (4.2%)	
**CLIP stage**					0.065			0.499
0	13 (14.9%)	7 (26.9%)	6 (9.8%)			3 (20.0%)	10 (13.9%)	
1	46 (52.9%)	14 (53.9%)	32 (52.5%)			9 (60.0%)	37 (51.4%)	
≥ 2	28 (32.2%)	5 (19.2%)	23 (37.7%)			3 (20.0%)	25 (34.7%)	
**BCLC classification**					0.611			0.365
A	26 (29.9%)	9 (34.6%)	17 (27.9%)			6 (40.0%)	20 (27.8%)	
B + C + D	61 (70.1%)	17 (65.4%)	44 (72.1%)			9 (60.0%)	52 (72.2%)	
**Infiltrative HCC**					1.000			1.000
No	80 (91.95%)	24 (92.3%)	56 (91.8%)			14 (93.3%)	66 (91.7%)	
Yes	7 (8.05%)	2 (7.7%)	5 (8.2%)			1 (6.7%)	6 (8.3%)	
Doxorubicin dosage (mg)	47.5 ± 23.7	48.7 ± 16.9	46.9 ± 26.2		0.144	46.6 ± 27.5	47.6 ± 23.1	0.657

^a^Mann-Whitney test was used for continuous variables and Fisher’s exact test was used for categorical variables.

^b^Mean ± standard deviation was presented.

^c^Median (inter-quartile range) was presented.

*Dox* doxorubicin, *HBV* hepatitis B virus, *HCV* hepatitis C virus, *ECOG* Eastern Cooperative Oncology Group, *CLIP* cancer of the liver Italian program, *BCLC* Barcelona Clinic Liver Cancer, *HCC* hepatocellular carcinoma.

### Safety

Patient biological variables including liver enzyme and bilirubin levels were compared before and after DEB-TACE treatment (Table 2). The levels of aspartate aminotransferase (AST), alanine aminotransferase (ALT), total bilirubin (T-bil), albumin, platelet (PLT), and creatinine (CRE) did not differ significantly between microsphere size or doxorubicin loading concentration.

**Table 2:** Deflection of biochemical indices in HCC patients (n = 87).

	Tandem size				*P*-value	Doxorubicin loading				*P*-value
	75 μm (n = 26)		100 μm (n = 61)			Half-loaded (n = 15)		Full-loaded (n = 72)		
	Before	After	Before	After		Before	After	Before	After	
**AST (U/L)**										
Mean ± SD	61.4 ± 51.4	78.5 ± 68.2	56.4 ± 32.5	107.6 ± 135.6		42.9 ± 20.6	50.2 ± 19.2	61.0 ± 41.1	109.0 ± 129.3	
Difference	17.1 ± 36.4		51.2 ± 133.2		0.547	7.3 ± 27.4		48.0 ± 123.8		0.120
**ALT (U/L)**										
Mean ± SD	50.6 ± 50.4	67.0 ± 61.8	41.2 ± 25.8	72.1 ± 91.4		38.3 ± 21.3	45.8 ± 31.6	45.2 ± 37.2	75.8 ± 89.7	
Difference	16.4 ± 29.5		30.9 ± 82.8		0.915	7.5 ± 24.9		30.6 ± 77.0		0.146
**T-bil (mg/dL)**										
Mean ± SD	0.8 ± 0.3	1.1 ± 0.5	0.9 ± 0.6	1.3 ± 1.1		1.0 ± 0.5	1.2 ± 0.7	0.9 ± 0.6	1.3 ± 1.0	
Difference	0.3 ± 0.4		0.4 ± 0.6		0.788	0.2 ± 0.4		0.4 ± 0.6		0.353
**Albumin (g/dL)**										
Mean ± SD	3.9 ± 0.5	3.8 ± 0.6	3.8 ± 0.6	3.8 ± 0.7		3.6 ± 0.4	3.5 ± 0.5	3.9 ± 0.6	3.9 ± 0.7	
Difference	− 0.1 ± 0.3		− 0.04 ± 0.6		0.187	− 0.1 ± 0.3		− 0.1 ± 0.5		0.566
**PLT (10**^**3**^**/μl)**										
Mean ± SD	121.5 ± 67.4	122.4 ± 65.5	148.6 ± 76.0	150.6 ± 75.7		132.6 ± 51.0	139.4 ± 70.9	142.2 ± 78.3	142.8 ± 74.6	
Difference	0.9 ± 51.4		2.0 ± 60.2		0.784	6.8 ± 57.0		0.6 ± 57.9		0.567
**CRE (mg/dL)**										
Mean ± SD	1.4 ± 1.9	1.3 ± 1.7	1.0 ± 0.6	1.0 ± 0.5		1.3 ± 0.6	1.3 ± 0.6	1.1 ± 1.2	1.0 ± 1.1	
Difference	− 0.04 ± 0.2		− 0.03 ± 0.2		0.897	− 0.04 ± 0.3		− 0.04 ± 0.2		0.629

Mann–Whitney test used to compare changes of values from baseline between different TANDEM size or different doxorubicin-loading dose.

*AST* aspartate aminotransferase, *ALT* alanine aminotransferase, *T-bil* Total bilirubin, *PLT* platelet, *CRE* creatinine.

Adverse events according to TANDEM size and doxorubicin-loading concentration are shown in Table 3. There were 11 (12.6%) patients, 36 (41.4%), and 36 (41.4%) patients experienced procedure-related complications, post-embolization syndrome and SIR classification complications, respectively. Most post-embolization syndrome were grade I or II, and most SIR classification complications were class A or B. A total of 46 adverse events was reported, of which pain (26.4%) and fever (19.5%) were the most common. Severe complications such as femoral artery pseudoaneurysm or prolonged hospitalization were not observed. Stratified of patients according to TANDEM size and doxorubicin-loading concentration revealed that those treated with 75-μm microspheres had a significantly higher rate of procedure-related complications than did those treated with 100-μm microspheres (26.9% vs 6.6%; P = 0.015). Patients treated with 75-μm microspheres were significantly more likely to have post-embolization syndrome of grade I (30.8% vs 21.3%) or grade II (26.9% vs 4.9%) than those treated with 100-μm microspheres (P = 0.016). Similarly, patients treated with 75-μm microspheres had a significantly higher rate of SIR class A (30.8% vs 21.3%) and class B (30.8% vs 4.9%) complications than those treated with 100-μm microspheres (P = 0.004). Postoperative pain was significantly more common in patients treated with 75-μm microspheres than in those treated with 100-μm microspheres (42.3% vs 19.7%; P = 0.036). On the other hand, patients treated with full-loaded doxorubicin microspheres had more procedure-related complications, post-embolization syndromes, and SIR class A/B complications than those treated with half-loaded doxorubicin microspheres, although the difference was not significant (P > 0.05). It is worth noting that 43 of 46 adverse events were observed in the full-loaded doxorubicin group, and 3 of 46 adverse events were observed in the half-loaded doxorubicin group. Patients treated with full-load doxorubicin microspheres had a significantly higher rate of fever than did those treated with half-load doxorubicin microspheres (P = 0.036). In summary, microspheres that are smaller (75 μm) and loaded to full capacity with doxorubicin have higher procedure-related complications, post-embolization syndromes, SIR class A/B complications, and adverse events.

**Table 3:** Adverse events and complications stratified by TANDEM size and doxorubicin-loading concentration.

	Total n (%)	TANDEM size			Doxorubicin loading		
		75 μm n (%)	100 μm n (%)	*P*-value^a^	Half-loaded n (%)	Full-loaded n (%)	*P*-value^a^
**Procedure-related complication**				0.015			0.199
No	76 (87.4%)	19 (73.1%)	57 (93.4%)		15 (100.0%)	61 (84.7%)	
Yes	11 (12.6%)	7 (26.9%)	4 (6.6%)		0 (0.0%)	11 (15.3%)	
**Post-embolization syndrome**				0.016			0.583
0	51 (58.6%)	10 (38.5%)	41 (67.2%)		12 (80.0%)	39 (54.2%)	
1	21 (24.1%)	8 (30.8%)	13 (21.3%)		2 (13.3%)	19 (26.4%)	
2	10 (11.5%)	7 (26.9%)	3 (4.9%)		1 (6.7%)	9 (12.5%)	
3	4 (4.6%)	1 (3.9%)	3 (4.9%)		0 (0.0%)	4 (5.6%)	
4	1 (1.2%)	0 (0.0%)	1 (1.6%)		0 (0.0%)	1 (3.0%)	
**SIR Classification System for complications**				0.004			0.542
No	51 (58.6%)	10 (38.5%)	41 (67.2%)		12 (80.0%)	39 (54.2%)	
A	21 (24.1%)	8 (30.8%)	13 (21.3%)		2 (13.3%)	19 (26.4%)	
B	11 (12.6%)	8 (30.8%)	3 (4.9%)		1 (6.7%)	10 (13.9%)	
C	3 (3.5%)	0 (0.0%)	3 (4.9%)		0 (0.0%)	3 (4.2%)	
D	1 (1.2%)	0 (0.0%)	1 (1.6%)		0 (0.0%)	1 (1.4%)	
**Adverse events**							
Abdominal pain	23 (26.4%)	11 (42.3%)	12 (19.7%)	0.036	3 (20.0%)	20 (27.8%)	0.750
Fever	17 (19.5%)	5 (19.2%)	12 (19.7%)	1.000	0 (0.0%)	17 (23.6%)	0.036
Acute gastritis	1 (1.2%)	1 (3.9%)	0 (0.0%)	0.299	0 (0.0%)	1 (1.4%)	1.000
Reflux esophagitis	1 (1.2%)	1 (3.9%)	0 (0.0%)	0.299	0 (0.0%)	1 (1.4%)	1.000
Nausea	3 (3.5%)	1 (3.9%)	2 (3.3%)	1.000	0 (0.0%)	3 (4.2%)	1.000
Liver abscess	1 (1.2%)	0 (0.0%)	1 (1.6%)	1.000	0 (0.0%)	1 (1.4%)	1.000

### Efficacy

Data regarding the tumor response show that 23 patients achieved complete response (CR), 32 achieved a partial response, 18 had stable disease, and 14 had progressive disease (Table 4). The mean hospital stay was 3.4 ± 2.2 days. No significant difference in the treatment response was observed between patients treated with 75- or 100-μm TANDEM microspheres (P = 0.602). However, patients treated with the 100-μm microspheres had significantly longer hospital stays than did those treated with the 75-μm microspheres (3.7 ± 2.3 vs. 2.8 ± 1.9 days; P, 0.023). On the other hand, patients treated with half-loaded doxorubicin microspheres had a significantly higher CR rate (53.3% vs 20.8%) and significantly shorter average hospital stay (1.7 ± 1.4 vs 3.8 ± 2.2 days, P < 0.001) than did those treated with full-loaded doxorubicin microspheres.

**Table 4:** Treatment response and length of hospital stay after treating with different TANDEM size or doxorubicin-loading dose.

Clinical outcomes	Total (n = 87)	TANDEM size			Doxorubicin loading		
		75 μm (n = 26)	100 μm (n = 61)	*P*-value^a^	Half-loaded (n = 15)	Full-loaded (n = 72)	*P*-value^a^
**Treatment response**				0.602			0.021
CR	23 (26.4%)	7 (26.9%)	16 (26.2%)		8 (53.3%)	15 (20.8%)	
PR	32 (36.8%)	11 (42.3%)	21 (34.4%)		3 (20.0%)	29 (40.3%)	
SD	18 (20.7%)	6 (23.1%)	12 (19.7%)		4 (26.7%)	14 (19.4%)	
PD	14 (16.1%)	2 (7.7%)	12 (19.7%)		0 (0.0%)	14 (19.4%)	
Length of hospital stay (days)	3.4 ± 2.2	2.8 ± 1.9	3.7 ± 2.3	0.023	1.7 ± 1.4	3.8 ± 2.2	< 0.001

Tumor response was evaluated 3 months after TACE treatment.

*CR* complete response, *PR* partial response, *SD* stable disease, *PD* progressive disease.

To further examine whether TANDEM size and doxorubicin-loading concentration are independent factors associated with better treatment response and length of hospital stay, multivariate regression analyses were performed by adjusting factors, including age, gender, CLIP stage, Child–Pugh Score, and total doxorubicin dosage. As shown in Table 5, patients treated with half-load doxorubicin microspheres had a higher likelihood of better treatment response (OR = 4.00, 95% CI = 1.06–15.13, ; P = 0.041) and shorter hospital stay (OR = − 1.72, 95% CI = − 2.77–0.68; P = 0.001) than those treated with full-load doxorubicin microspheres (Table 5). Moreover, there was no statistically significant difference in the better treatment response or length of hospital stay between treatment with 75- and 100-μm microspheres.

**Table 5:** Adjusted association of TANDEM size and drug-loading concentration with clinical outcomes and biochemical measures in patients with hepatocellular carcinoma.

Outcomes	TANDEM size (ref = 100 μm)	*P*-value	Doxorubicin loading (ref = Full loaded)	*P*-value
	Estimate (95% CI)		Estimate (95% CI)	
Better treatment response^a^	0.99 (0.30, 3.31)	0.986	4.00 (1.06, 15.13)	0.041
Length of hospital stay (days)^b^	− 0.84 (− 1.71, 0.02)	0.056	− 1.72 (− 2.77, -0.68)	0.001

^a^Logistic regression was performed after adjusting for age, gender, CLIP stage, Child–Pugh Score, and total doxorubicin dosage. Odds ratio and 95% CI were presented.

^b^Linear regression was performed after adjusting for age, gender, CLIP stage, Child–Pugh Score, and total doxorubicin dosage. The regression coefficients (β) indicated the difference in days between TANDEM size or doxorubicin-loading groups.

Stratified analysis of doxorubicin-loading concentration further showed that in patients treated with 100-μm microspheres, a half-load doxorubicin resulted in higher CR (63.6% vs 18.0%) and ORR (90.9 vs 54.0%) than did full-loaded doxorubicin. In contrast, in patients treated with 75-μm microspheres, full-load doxorubicin showed a higher ORR (77.3% vs 25%) than did a half-load doxorubicin (Table 6). On the other hand, a shorter hospital stay was significantly associated with half-loaded doxorubicin microspheres, regardless of their size (P < 0.001). Notably, patients treated with 100-μm microspheres carrying a half-load of doxorubicin had the shortest average hospital stay (1.6 ± 1.3 days).

**Figure 1:**
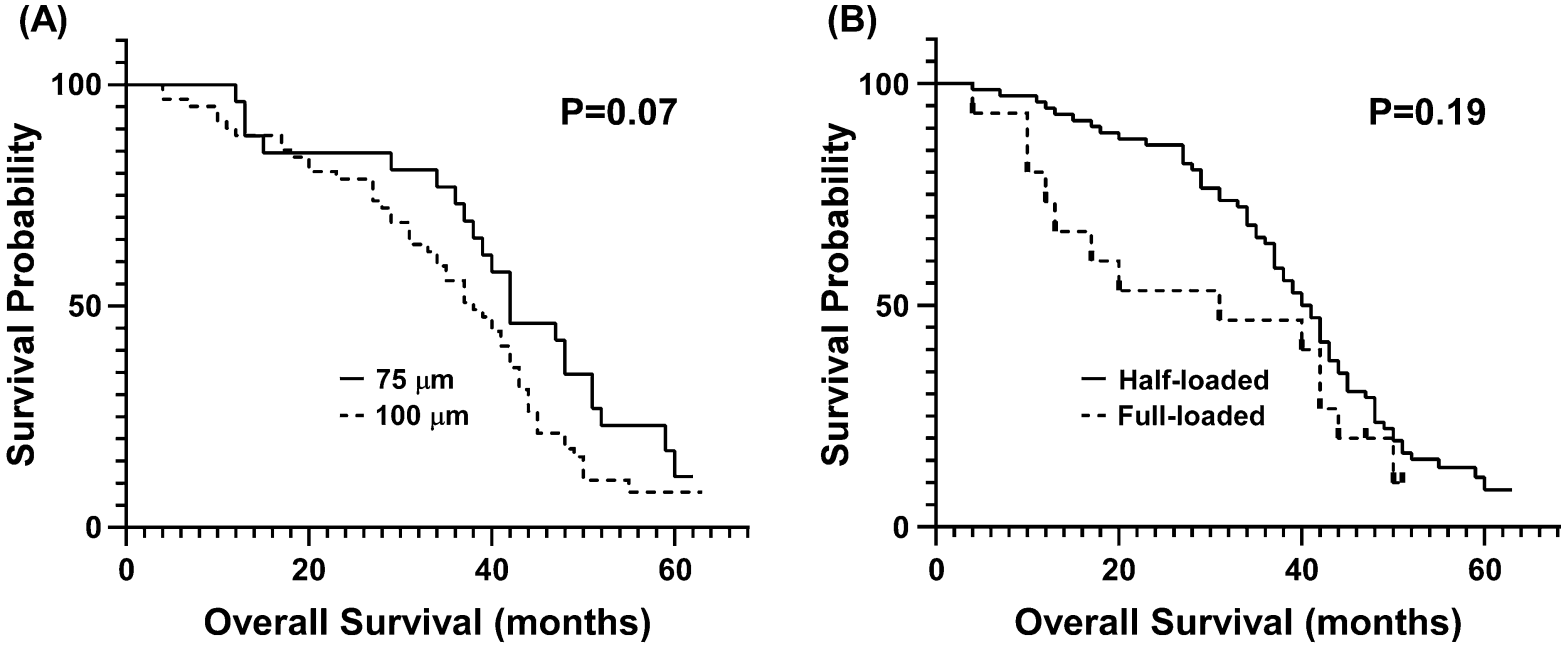
Kaplan–Meier survival curve of hepatocellular carcinoma patients who underwent DEB-TACE with (**A**) 75-µm or 100-µm microspheres or (**B**) with half-load or full-load doxorubicin microspheres.

The Kaplan–Meier survival curve of HCC patients stratified by microsphere size and doxorubicin loading concentration is shown in Fig. 1. No significant difference in the median OS was observed between patients receiving 75- and 100-µm microspheres (42 vs. 38 months, respectively) (P > 0.05) or between those receiving half- and full-load doxorubicin microspheres (31 vs. 40 months, respectively) (P > 0.05). Cox proportional hazards regression analysis revealed no difference in OS between microsphere size and doxorubicin loading concentration (P > 0.05) (Table 7).

**Table 6:** Association of microsphere size and doxorubicin-loading concentration with overall survival in HCC patients.

	Univariate		Multivariate^a^	
	HR (95% CI)	*P*-value	aHR (95% CI)	*P*-value
**TANDEM microsphere size**				
75 μm	0.64 (0.38, 1.05)	0.077	0.68 (0.39, 1.19)	0.175
100 μm	Ref		Ref	
**Doxorubicin-loading concentration**				
Half-loaded	1.48 (0.81, 2.69)	0.205	1.67 (0.88, 3.16)	0.117
Full-loaded	Ref		Ref	

^a^Cox proportional hazards regression analysis was performed with adjustment for age, gender, CLIP stage, Child–Pugh Score, and total doxorubicin dosage.

*HR* hazard ratio, *aHR* adjusted hazard ratio, *CI* confidence interval, *ref* reference.

## Discussion

Although previous studies have shown that small-diameter microspheres are safe and effective in DEB-TACE HCC treatment^41–43^, the safety and efficacy correlation between microspheres size ≤ 100 µm and the loading concentration of anticancer drug remain unclear. In the present study, we found that the size and doxorubicin-loading concentration of TANDEM microspheres did not affect the OS, but significantly correlated with the treatment response and complications in HCC patients. Larger microsphere size (100 µm) and lower doxorubicin loading (half-loaded) showed a trend toward fewer procedure-related complications, post-embolization syndrome, SIR complications, and abdominal pain and fever. In addition, half-load doxorubicin microspheres correlated significantly with better treatment outcomes and shorter hospital stays. Stratified analysis further showed that treatment using 100-µm microspheres with half-loaded doxorubicin had better outcomes and shorter hospital stays. These results suggest that while microsphere size and doxorubicin-loading concentration may not affect survival outcomes, the use of 100-µm microspheres loaded with lower doxorubicin concentration may decrease procedure-related complication, post-embolization syndrome, and SIR complication, as well as decrease the length of hospitalization.

The findings of a liver tumor study in rabbits by Tanaka et al.^26^ support the results of the present study. At the same concentration of irinotecan (50 mg/mL), low-dose irinotecan (0.5 mg/kg) had a lower histological tumor necrosis rate than did high-dose irinotecan (1 mg/kg). The rates of tumor necrosis for low- and high-dose irinotecan on day 7 were 65% and 90%, respectively. However, a 50% reduction in the irinotecan concentration (25 mg/mL; 0.5 mg/kg)resulted in a higher tumor necrosis rate in the high total dose/concentration group (100% for 25 mg/mL; 0.5 mg/kg vs. 90% for 50 mg/mL; 1 mg/kg). Similarly, our results show that regardless of bead size, half-load doxorubicin microspheres had a better treatment outcome (OR, 4.00; P < 0.05) and shorter hospital stays (OR, − 1.72; P, 0.001) than did full-load doxorubicin microspheres.

Overall, treatment with 100-µm half-load doxorubicin microspheres resulted in the best treatment response (Table 6). These results may be attributable to the influence of doxorubicin loading on the physical properties and drug distribution in the microsphere beads. An increase in the amount of doxorubicin loaded is accompanied by a decrease in the water content of the beads, leading to a decrease in the average bead diameter and an increase in the resistance of compression force of the beads^44^. In other words, loading half the maximum dose of doxorubicin into microspheres does not greatly reduce its equilibrium water content. In contrast to cTACE, a slow release of the chemotherapeutic agent is important in DEB-TACE, and the size of the microspheres is an important determinant of the elution rate. Lewis et al. showed that doxorubicin is not uniformly distributed inside the microspheres but rather is concentrated in the 20-µm outer layer of the beads^44^. Smaller-diameter beads have a larger relative surface area than larger beads, resulting in faster elution of the chemotherapeutic agent^45,46^. Increasing the loading concentration does not prolong the drug release time and only increases the maximum plasma concentration^30^. In addition, reducing the drug loading concentration in drug-eluting beads has the advantage of reducing complications and adverse events. Thus, in addition to lower complications and adverse events, 100-µm microspheres loaded with a half dose of doxorubicin had the best treatment outcomes (ORR = 90.9%) and the shortest hospital stay (1.6 days). However, these results may not be representative of all microspheres. Kalva et al. observed that 100–300 µm LC Bead loaded with 25 mg/mL doxorubicin resulted in only 2.5% CR and 8.7% PR^47^. In addition, loading capacity and efficiency differ between drugs and bead types. Therefore, for other types of microspheres and drugs, more in-depth studies are needed to explore the correlation between microsphere size and optimal drug loading.

**Table 7:** Statistics of treatment response and length of hospital stay stratified by both TANDEM size and drug-loading concentration.

	TANDEM size					*P*-value^**a**^
	75 μm			100 μm		
	Half-loaded (Group 1, n = 4)		Full-loaded (Group 2, n = 22)	Half-loaded (Group 3, n = 11)	Full-loaded (Group 4, n = 50)	
**Treatment response**						0.020
CR	1 (25.0%)		6 (27.3%)*	7 (63.6%)	9 (18.0%)‡	
PR	0 (0.0%)		11 (50.0%)	3 (27.3%)	18 (36.0%)	
SD	3 (75.0%)		3 (13.6%)	1 (9.1%)	11 (22.0%)	
PD	0 (0.0%)		2 (9.1%)	0 (0.0%)	12 (24.0%)	
**ORR**						0.016
Yes (CR + PR)	1 (25.0%)		17 (77.3%)	10 (90.9%)*	27 (54.0%)‡	
No (SD + PD)	3 (75.0%)		5 (22.7%)	1 (9.1%)	23 (46.0%)	
**DCR**						0.144
Yes (CR + PR + SD)	4 (100.0%)		20 (90.9%)	11 (100.0%)	38 (76.0%)	
No (PD)	0 (0.0%)		2 (9.1%)	0 (0.0%)	12 (24.0%)	
Length of hospital stay (days)	2.0 ± 2.0		3.0 ± 1.9	1.6 ± 1.3	4.2 ± 2.3^**†,‡**^	< 0.001

Treatment response, ORR and DCR were resented as number and percentage.

Length of hospital stay were presented as mean ± SD (days).

^a^Fisher's exact test and Kruskall-Wallis test was performed for treatment response and length of hospital stay, respectively.

*P < 0.05 compared to Group 1 by Fisher's exact test for treatment response and Mann–Whitney test for length of hospital stay.

^†^*P* < 0.05 compared to Group 2 by Fisher's exact test for treatment response and Mann–Whitney test for length of hospital stay.

^‡^*P* < 0.05 compared to Group 3 by Fisher's exact test for treatment response and Mann–Whitney test for length of hospital stay.

*CR* complete response, *PR* partial response, *SD* stable disease, *PD* progressive disease, *ORR* objective response rate, *DCR* disease control rate.

Several studies report a decrease in platelet count after TACE^48,49^. In contrast, we observed no significant decrease in the platelet count in our patients. In addition, the post-TACE values of T-bil, albumin, and creatinine remained within the normal range. Liver enzyme levels are known to increase after TACE treatment. In this study, the post-TACE AST and ALT levels increased significantly. Notably, the changes in AST and ALT levels were significantly higher in patients treated with full-load than half-load doxorubicin microspheres (AST: 48.0 U/L vs. 7.3 U/L; ALT: 30.6 U/L vs. 7.5 U/L). The lack of statistical significance for these data may result from our small sample size. Although the finding did not reach statistical significance, half-load doxorubicin microspheres may cause less liver damage than full-loaded doxorubicin microspheres.

This study has several limitations. First, the safety and efficacy of only two sizes of TANDEM microspheres and two loading doxorubicin concentrations were investigated in a small number of HCC patients. Because the sample size is not large enough, although half-load doxorubicin microspheres seems to be associated with fewer adverse events, lower complication, and smaller changes in liver function, the differences are not statistically significant. Second, since this study is retrospective, data regarding the maximum plasma concentration (C_max_) and area under the curve (AUC) of doxorubicin were not available. In addition, the lack of data regarding patient progression in this retrospective cohort precluded calculation of the progression-free survival after treatment. Despite the promising results of our study, further prospective studies are needed that are of randomized controlled design, include more participants, compare the C_max_ and AUC of doxorubicin in different sizes of microspheres and at different loading concentrations, and can determine survival outcomes by conducting long-term follow-up.

## Conclusions

This study indicates that both 75- and 100-μm TANDEM microspheres are safe and effective for HCC treatment. Although the OS was not affected by the size or doxorubicin loading concentration of the microspheres, these parameters significantly affected the rates of treatment response, adverse events, complications and hospitalization length. Although this study shows promising results, further randomized control trials are required to verify the findings and determine the optimal doxorubicin loading concentration for treating HCC patients.
